# Forest Gaps Inhibit Foliar Litter Pb and Cd Release in Winter and Inhibit Pb and Cd Accumulation in Growing Season in an Alpine Forest

**DOI:** 10.1371/journal.pone.0131528

**Published:** 2015-06-26

**Authors:** Jie He, Wanqin Yang, Han Li, Liya Xu, Xiangyin Ni, Bo Tan, Yeyi Zhao, Fuzhong Wu

**Affiliations:** Key laboratory of Ecological Forestry Engineering, Long-term Research Station of Alpine Forest Ecosystem, Institute of Ecology and Forestry, Sichuan Agricultural University, Chengdu 611130, China; Technical University in Zvolen, SLOVAKIA

## Abstract

**Aims:**

The release of heavy metals (such as Pb and Cd) from foliar litter play an important role in element cycling in alpine forest ecosystems. Although natural forest gaps could play important roles in the release of heavy metals from foliar litter by affecting the snow cover during the winter and solar irradiation during the growing season, few studies have examined these potential roles. The objectives of this study were to document changes in Pb and Cd dynamics during litter decomposition in the center of gaps and under closed canopies and to investigate the factors that controlled these changes during the winter and growing seasons.

**Methods:**

Senesced foliar litter from six dominant species, including Kangding willow (*Salix paraplesia*), Masters larch (*Larix mastersiana*), Mingjiang fir (*Abies faxoniana*), Alpine azalea (*Rhododendron lapponicum*), Red birch (*Betula albosinensis*) and Mourning cypress (*Sabina saltuaria*), was placed in litterbags and incubated between the gap center and closed canopy conditions in an alpine forest in the eastern region of the Tibetan Plateau. The litterbags were sampled at the snow formation stage, snow coverage stage, snow melt stage and during the growing season. The Pb and Cd concentrations in the sampled foliar litter were determined by acid digestion (HNO_3_/HClO_4_).

**Important findings:**

Over one year of decomposition, Pb accumulation and Cd release from the foliar litter occurred, regardless of the foliar litter species. However, Pb and Cd were both released from the foliar litter during the winter and accumulated during the growing season. Compared with the gap center and the canopy gap edge, the extended gap edge and the closed canopy showed higher Pb and Cd release rates in winter and higher Pb and Cd accumulation rates during the growing season, respectively. Statistical analyses indicate that the dynamics of Pb were significantly influenced by frequent freeze–thaw cycles in winter and appropriate hydrothermal conditions during the growing season, the dynamics of Cd were strongly influenced by species and the presence of a forest gap at different decomposition stages. These results show that forest gaps could inhibit Pb and Cd release from foliar litter in the alpine forest of western Sichuan. In addition, a decrease in the snow depth in the winter warming scenario would promote the release of Pb during foliar litter decomposition. There exist some difference that may be influenced by litter quality, microenvironment and microtopography during litter decomposition.

## Introduction

The amount of certain elements delivered to the soil by decomposing litter is an important component of sustainable forest management [1]. Among potentially toxic elements, lead (Pb) and cadmium (Cd) have been considered among the top 20 pollutants on the USEPA priority list [2]. These heavy metals are toxic to most soil organisms when present in excessive concentrations[3], and the toxicity to soil organisms has a negative impact on material circulation and energy transformation of alpine forest ecosystem. Canopy gaps are recognized as important determinants of forest regeneration [4, 5], and gap formation by disturbances can alter the snow thickness, light intensity, and other environmental conditions that may influence the temporal and spatial dynamics of Pb and Cd at different scales. In our previous studies of foliar litter decomposition, the seasonal snow cover had a significant impact on mass loss, nutrient dynamics and microbial biomass in the alpine forest [6–8]. Although heavy metal dynamics have frequently been studied in forest ecosystems [9–11], data regarding the dynamics of Pb and Cd during litter decomposition in alpine regions, because of variation in the radiation and snow cover, particularly regarding the effects of forest gaps on Pb and Cd dynamics, remain relatively scarce.

Forest gaps affect the Pb and Cd dynamics during litter decomposition differently during the winter and growing seasons. Seasonal snow cover is one typical feature of high latitude/altitude ecosystems. Deep and moderate snow patches occur in the gap centers and at the canopy gap edges, which can provide relatively stable environments in which soil organisms can thrive [12]. In addition, the insulating effects of snow and snowmelt result in moisture-temperature conditions that are favorable for the decomposition of foliar litter. As the temperature increases, snowmelt occurs during the snowmelt stage, which results in Pb and Cd leaching [13]. The aforementioned factors may induce the release of Pb and Cd during litter decomposition. Compared with gap centers and canopy gap edges, the thin and absent snow patches at the extended gap edge and under the closed canopy display more intense freezing and repeated freeze-thaw cycles, which can damage the physical structure of the litter and improve litter decomposition [14, 15]. Previous studies have indicated that litter quality may influence decomposition [16–18]. Freeze-thaw cycles result in significant changes in litter chemistry by affecting litter physical structure and improving litter decomposability [19, 20] and potentially contribute to Pb and Cd release from foliar litter. Canopy gap disturbance can influence the availability of resources, such as light, water, and nutrients during the growing season [21]. Since the composition and abundance of the understory are different, the light intensities are greatest in the gap centers, where direct irradiation reaches the forest floor and increases the soil temperature and decreases the soil moisture content relative to other locations. In addition, the nutrient contents in the gap varied widely from the gap center to the closed canopy [22]. Therefore, the forest gaps can be an important factor for controlling microbial activity and, consequently, litter decomposition and the biogeochemical cycling of Pb and Cd.

There is snow insulating effect in the gap during the winter and a canopy insulating effect in the closed forest during the growing season. The extended gap edge and closed canopy provide the soil organisms with microenvironments that are suitable for their activities [23], thereby improving Pb and Cd release or immobilization during litter decomposition through biological processes during the growing season. The bioavailability of Pb is generally lower relative to Cd, confirming that this metal does not readily remain in the soluble fraction [2, 24]. Consequently, Pb release would be more difficult than that of Cd. Moreover, the Cd mobility in a soil depends more strongly on the acidity, whereas that of Pb is controlled by the concentration of organic substances [25], which may result in different amounts of Pb and Cd release. However, the research on Pb and Cd dynamics during litter decomposition is insufficient. The objective of this study was to characterize the effects of forest gaps on Pb and Cd dynamics during decomposition of litter from six representative woody species in different snow cover stages and during the growing season in an alpine forest in western Sichuan. Therefore, we hypothesized the following: 1) more Pb and Cd are released from decomposing litter in the gap center than under the closed canopy because of higher decomposer activity under snow cover and higher leaching during snowmelt, 2) more Pb and Cd accumulate in foliar litter under the closed canopy due to higher decomposers quantity and activity in the growing season, and 3) Cd release occurs more readily than Pb release.

To test these hypotheses, a field experiment using the litterbag method was conducted in an alpine forest located on the eastern edge of Qinghai–Tibet Plateau and the upper reaches of the Yangtze River from November 2012 to October 2013 with intermediate sampling dates. This region plays an important role in conserving soil and water, preserving biodiversity, regulating climate and improving the environment [26]. On the one hand, heavy metals in the Yangtze River have relative high concentration because of parental rocks. The release of heavy metal from litter in upper reaches could aggravate the potential pollution. On the other hand, heavy metals are also the important elements in plant organisms, although they are not necessary for plant growth. The cycling of heavy metals is also one of the essential processes in forest ecosystems, but lack of attention.

## Materials and Methods

### Ethics Statement

The Institute of Ecological Forestry, Sichuan Agricultural University, received a permit from the Western Sichuan Forestry Bureau to conduct scientific experiments in the Bipenggou Nature Reserve since March 2006. The senescent fresh foliar litter collected for this study were only sampled at a very limited scale, and thus had negligible effects on broader ecosystem functioning. Moreover, this research was carried out in compliance with the laws of the People's Republic of China. The research did not involve measurements of humans or animals and no endangered or protected plant species were involved.

### Study sites

The study area is located in the Miyaluo Nature Reserve in Lixian County, Sichuan, China (102°53′–102°57′E, 31°14′–31°19′N, 2458–4619 m a.s.l.). This region is a transitional area between the Qinghai–Tibet Plateau and the Sichuan Basin. The annual mean air temperature is 3°C, the coldest month is January (-18°C), and the warmest month is July (23°C). The annual mean precipitation is approximately 850 mm, with most rainfall occurring between May and August. During the freeze-thaw season, the soil remains frozen for 5–6 months, and obvious and frequent freeze-thaw cycles occur during the onset of freezing and thawing [27]. The forest vegetation in the study region consists of deciduous broadleaf forest, mixed coniferous and broadleaf forest, coniferous forest, alpine shrub, and alpine meadow along an altitudinal gradient [28]. The forest is dominated by Masters larch (*Larix mastersiana*), Mingjiang fir (*Abies faxoniana*), Red birch (*Betula albosinensis*) and Mourning cypress (*Sabina saltuaria*), interspersed with shrubs of Kangding willow (*Salix paraplesia*) and Alpine azalea (*Rhododendron lapponicum*).

### Experimental design

Our study site consisted of 72 experimental plots (3 gaps×4 locations×6 plots) that were located in three replicate gaps (larger than 25×25 m) at an elevation of 3,582 m in a fir-dominated forest. According to Schliemann & Bockheim [29], We definite canopy gap: a ‘hole’ in the forest through all levels down to an average height of 2 m above ground, and extended gap: canopy gap plus the area that extends to the bases of surrounding canopy trees. Gap age may be determined from tree ring analysis of released trees in or near the gap edge. The gaps here we selected are natural formed about 20 years ago [30]. Some decayed stumps and trunks are standing on the forest floor. We have not changed any characters of soil surface or vegetation. Four locations were identified along the gradient from a forest gap to full canopy cover in each gap, the gap center, canopy gap edge, extended gap edge and closed canopy. Six 2×2 m experimental plots for six litter species in each location, with a distance greater than 3–4 m between the two adjacent locations.

**Fig 1:**
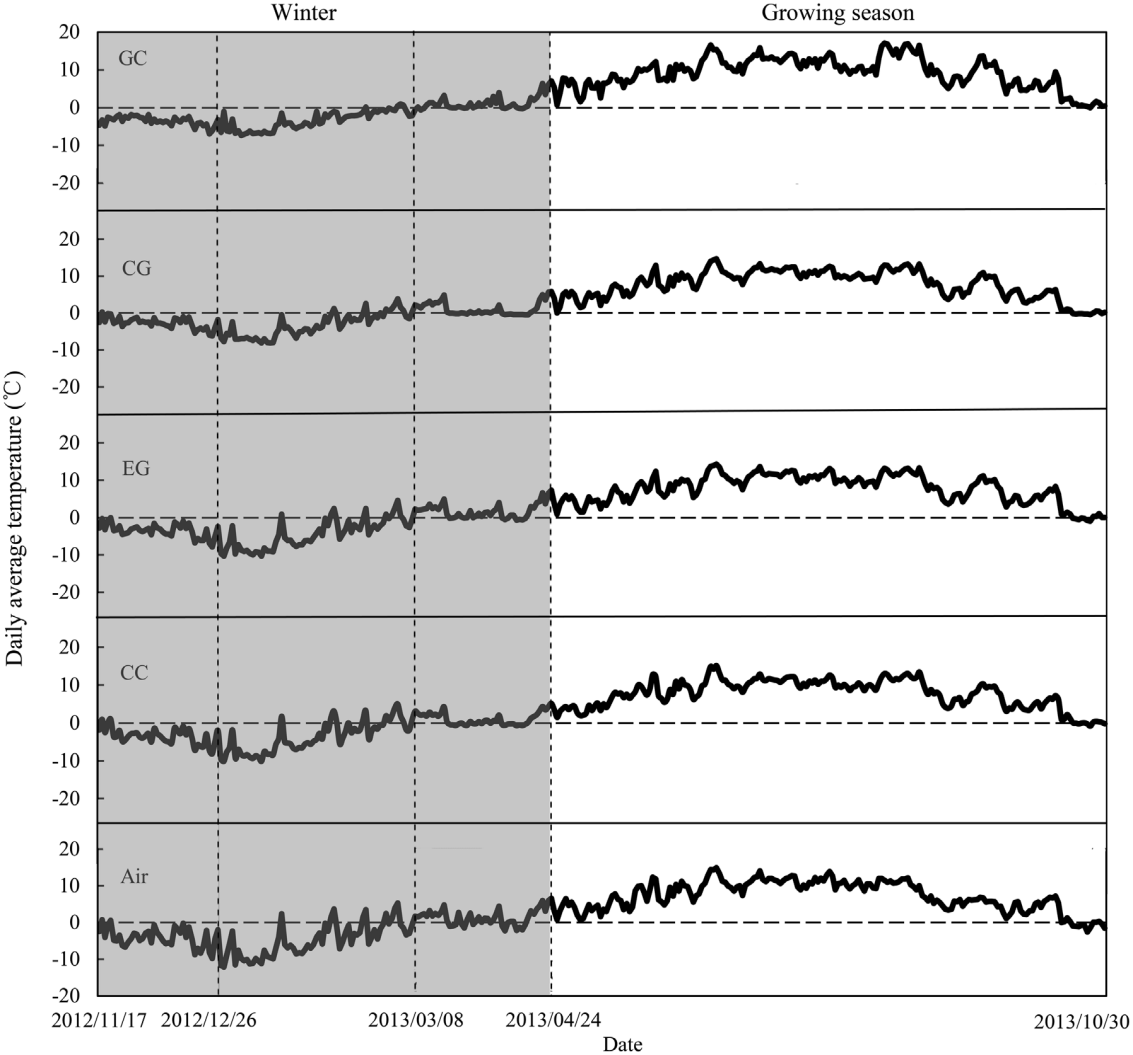
The dynamics of litter and air temperature under different snow cover conditions in alpine forest of western Sichuan. GC, gap center; CG, canopy gap edge; EG, extended gap edge; CC, closed canopy. The shaded area shows the temperature dynamics in winter.

**Fig 2:**
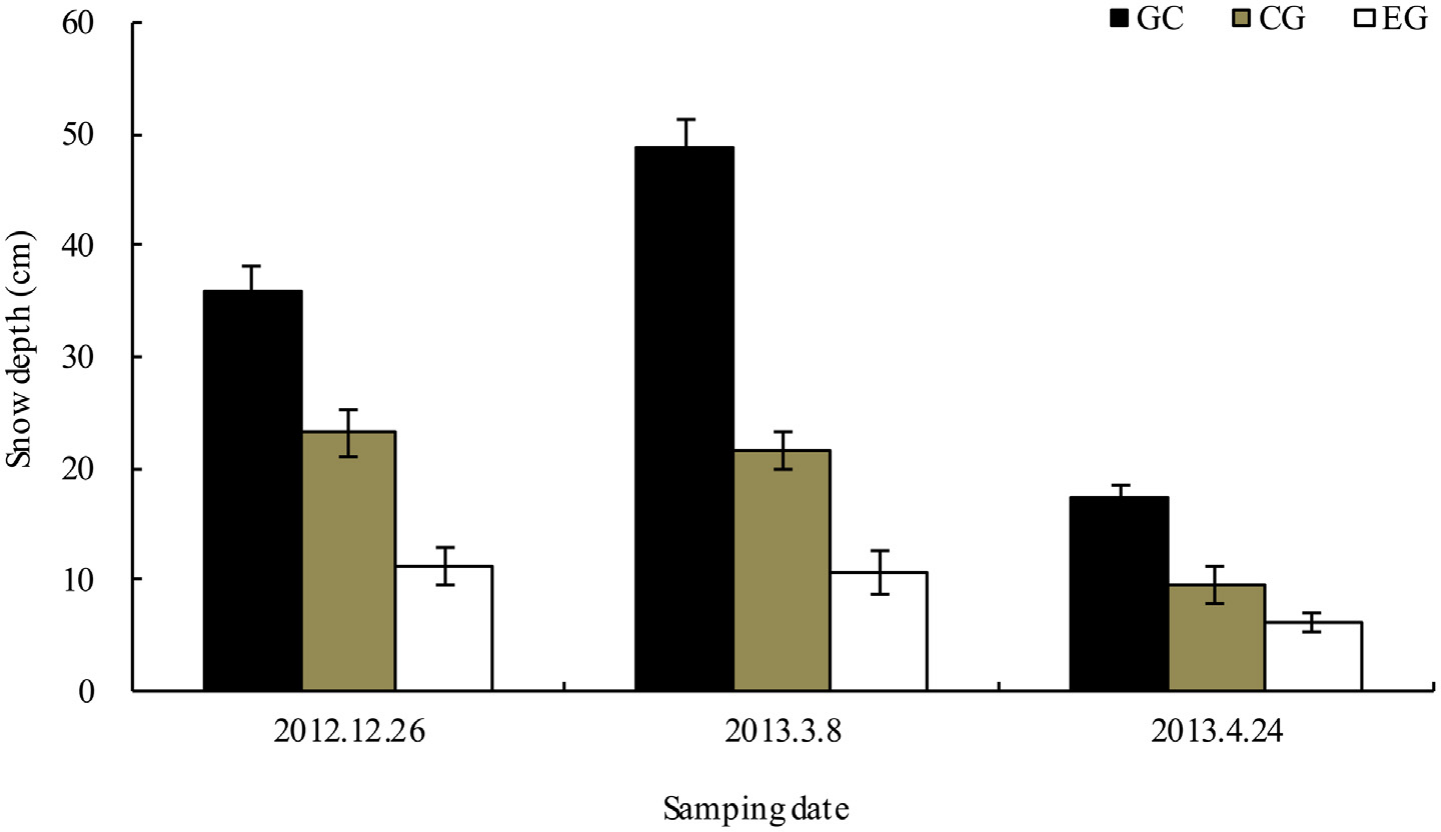
Thickness changes of snow cover in different stages (mean ± SD). GC, gap center; CG, canopy gap; EG, extended gap.

The litter fall occurs at least one month between autumn and winter in the studied alpine forest. In October 2012, freshly senesced willow, larch, fir, azalea, birch and cypress leaves were collected from the floor of the sampled forests. These species we selected have similar litterfall time. Air-dried litter samples (10 g per bag) were placed in nylon bags (20×25 cm with 0.5 mm (top) and 1.0 mm (bottom) mesh), and the edges were sealed. Overall, 144 litterbags of each species (three gaps×four plots×four stages×three replicates) were placed on the floors of the three sampled forests on November 15, 2012. At least 2 cm was maintained between consecutive bags to avoid disturbance. Five replicate samples for each species were used to determine the initial litter quality (Table 1). In the meantime, the iButton DS1923-F5 recorders (Maxim/Dallas Semiconductor, Sunnyvale, CA, USA) that recorded soil temperature every 2 h were placed in two litterbags in each location. The litter and air temperature dynamics are described in Fig 1. Across the forest gaps, we observed the formation of snow patches of different thicknesses due to canopy cover, wind and other factors. Snow thickness was measured with a ruler at multiple points on each sampling date (Fig 2).

**Table 1:** Initial concentrations of carbon (C), nitrogen (N), phosphorus (P) (g·kg-1 dry weight) and metals concentrations (mg·kg-1 dry weight) for foliar litter (mean ± standard deviation, n = 3).

Species	C	N	P	Pb	Cd
Fir	505.60±29.62a	8.75±0.03a	1.14±0.10ad	0.90±0.16a	0.06±0.01ae
Cypress	516.37±17.68a	8.77±0.10a	1.24±0.06ab	0.98±0.11a	0.12±0.01b
Larch	543.49± 6.30a	8.60±0.41a	1.33±0.02b	0.91±0.03a	0.10±0.02ab
Birch	496.86±14.52ab	13.34±0.22b	0.91±0.04c	1.96±0.12b	0.61±0.06c
Willow	452.27±16.52b	11.46±0.89c	1.11±0.02d	1.60±0.07c	1.53±0.03d
Azalea	502.91±15.98a	6.66±0.20d	1.07±0.09d	0.94±0.16a	0.03±0.01e

Different lowercase letters in the same column mean significant differences (p < 0.05) among species.

Litterbags were sampled at four stages: the snow formation stage, snow coverage stage, snowmelt stage, and growing season. Three replicate litterbags for each plot and species were randomly sampled from each plot on December 26, 2012, March 8, 2013, April 24, 2013, and October 30, 2013. Each date represented one of the four stages.

### Analyses and calculations

The residual litter in the litterbags after thoroughly cleaning out debris was oven-dried to a constant weight at 65°C for at least 72 h before grinding and passing through a 0.3-mm stainless steel sieve for chemical analysis. For the Pb and Cd analysis, approximately 0.50 g of dry litter material was digested with a mixture of HNO_3_ and HClO_4_ (5:1). Next, the digested solution was transferred to a 50-ml volumetric flask, rationed, and stored for Pb and Cd concentration measurements. The Pb and Cd concentrations in the initial and decomposed leaves were determined using an inductively coupled plasma emission spectrophotometer (ICP-AES; Thermo Electron Corporation, Massachusetts, USA) [11, 31]. Organic carbon, total nitrogen and total phosphorus were determined using dichromate oxidation, Kjeldahl digestion and phosphomolybdenum-yellow spectrophotometry, respectively [32].

The percentage of metal release (*E*) was calculated as follows [33]:

$$E(\text{\%})=(C_{t-1}M_{t-1}-C_{t}M_{t})/C_{i}M_{i}\times 100$$

where *C*_*t-1*_ and *C*_*t*_ are the concentration of the metal at sampling time t-1 and t, respectively; *C*_*i*_ is the initial concentration of the metal (mg·kg^-1^); *M*_*t-1*_ and *M*_*t*_ are the dry mass of the decomposed leaf litter at time t-1 and t; *M*_*i*_ is the initial dry mass of the litter. The sum of the release rates during snow formation, snow cover and snowmelt is equal to the release rates of freeze-thaw season, and the sum of the release rates during the freeze-thaw season and growing season is equal to the release rates of 1 year. The positive values of E mean release, while negative values mean accumulation.

The metal released per day (*V*_*t*_) was calculated as follows [6]:

$$V_{t}(\%\;\text{released}/\text{day})=E(\text{\%})/\Delta t$$

where *Δt* is the day interval between two consecutive sampling periods.

### Statistical analysis

Differences in the initial substrate, Pb and Cd concentrations, release rates among locations were evaluated by using one-way ANOVA with Fisher’s LSD post hoc testing. We have examined the date for homogeneity of variances (Levene’s test) before analysis. The two-factor analysis of variance and LSD methods were used to compare the interactions between the gap and species with the Pb and Cd release rates from the litter. A Pearson's correlation coefficient test was performed to determine the relationships between temperature and Pb and Cd release rates. All statistical analyses were conducted using Excel 2003 and SPSS 20.0.

## Results

### Metal concentrations in the decomposing litter

**Fig 3:**
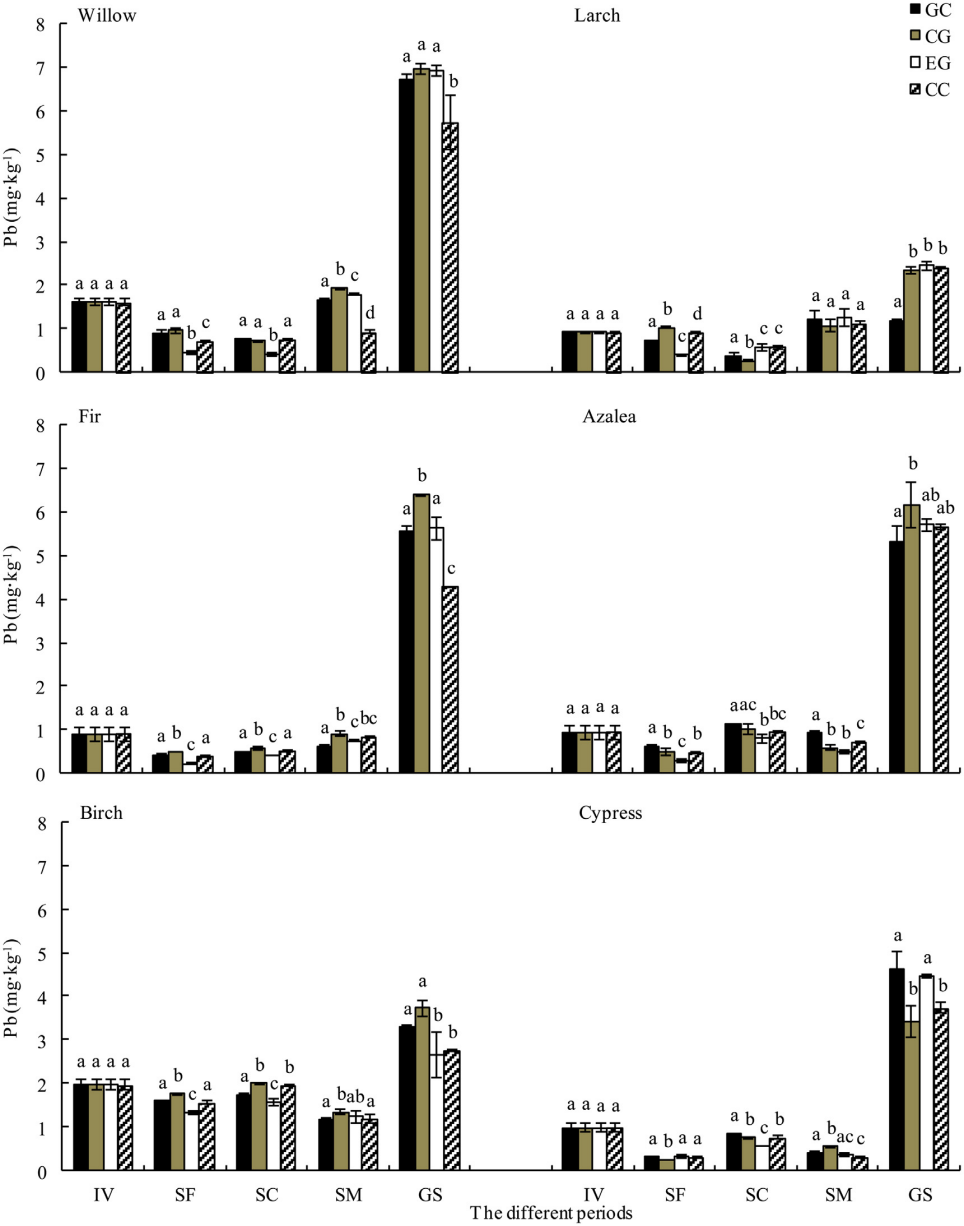
Dynamics of Pb concentration in litter during decomposition from gap center to closed canopy (mean ± *SD*, *n = 3*). IV, initial value; SF, snow formation stage; SC, snow coverage stage; SM, snow melt stage; GS, growing season. GC, gap center; CG, canopy gap; EG, extended gap; CC, closed canopy. Different lowercase letters indicate significant differences (*p* < 0.05) among different snow patches within the same stages.

The Pb concentration decreased at the snow formation stage, regardless of species (Fig 3). However, the Pb concentrations in the willow, larch and fir litter generally increased after the snow coverage stage, and the Pb concentrations in azalea, birch and cypress litter decreased at the snow melt stage (Fig 3). Large increases in Pb concentrations were observed regardless of the foliar litter species during the growing season (Fig 3). During each stage, the Pb concentrations in the willow, fir, azalea, birch and cypress litter were greater in the gap center compared with those in the closed canopy. However, the Pb concentrations in the larch litter were greater at the extended gap edge and under the closed canopy, except during the snow formation stage.

**Fig 4:**
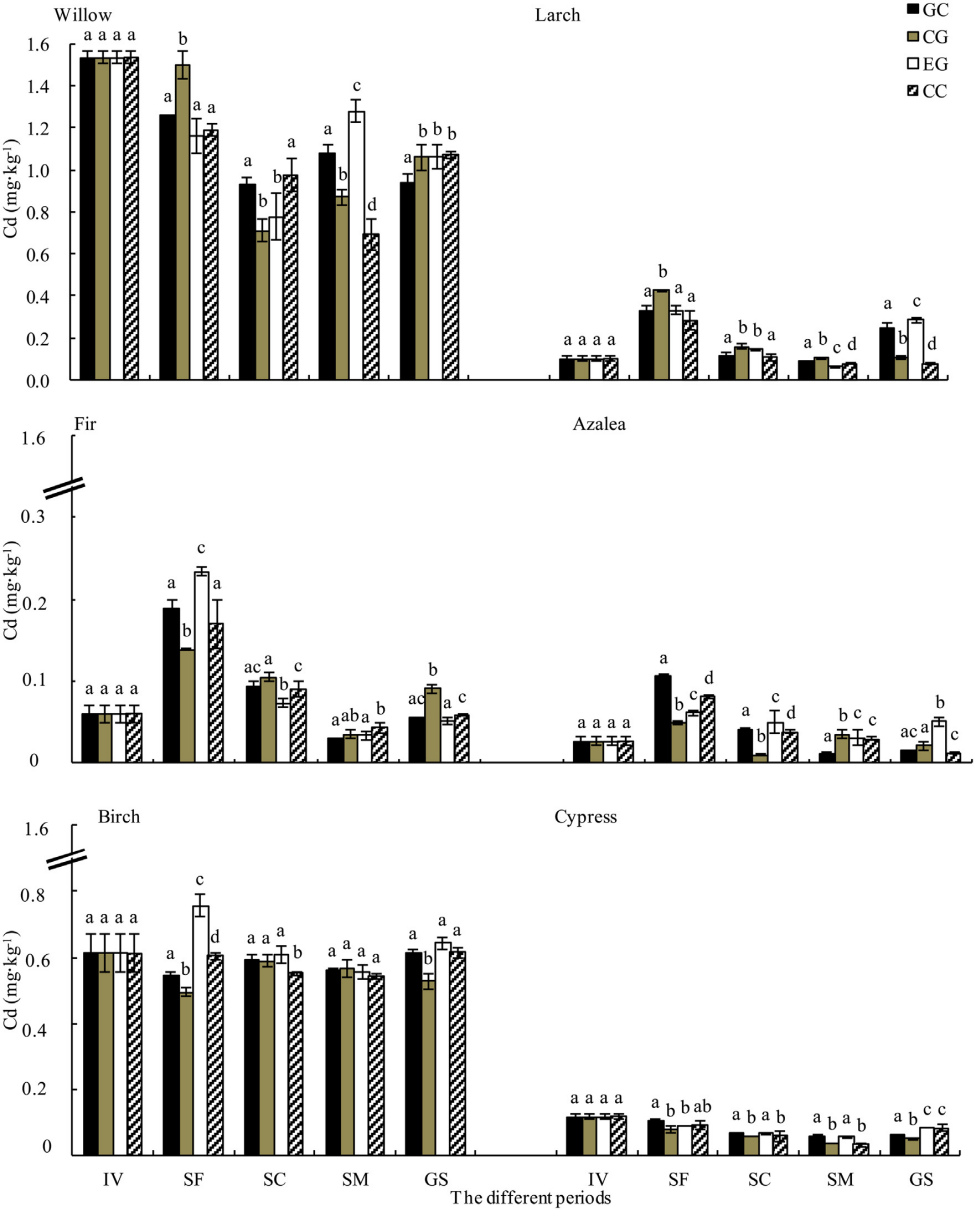
Dynamics of Cd concentration in litter during decomposition from gap center to closed canopy (mean ± *SD*, *n = 3*). See Fig 3 for notes.

The Cd concentrations in the larch, fir and azalea litter increased at the snow formation stage, decreased during the snowmelt stage, and quickly increased during the growing season (Fig 4). The Cd concentrations in the cypress litter gradually decreased with time until the snow melt stage (Fig 4). No significant changes were observed in the birch litter. In addition, the Cd concentrations increased regardless of species during the growing season (Fig 4). The Cd concentrations in the willow, larch, azalea and birch litter were greater in the gap center and at the canopy gap edge during the snow formation stage. During the snow coverage stage, the Cd concentrations in the larch, fir and cypress litter were higher in the gap center and at the canopy gap edge. The Cd concentrations in the willow and fir litter were greater at the extended gap edge and under the closed canopy at the snowmelt stage. The Cd concentrations in the litter, excluding fir, were greater at the extended gap edge and under the closed canopy during the growing season.

### Metal release during decomposition

**Fig 5:**
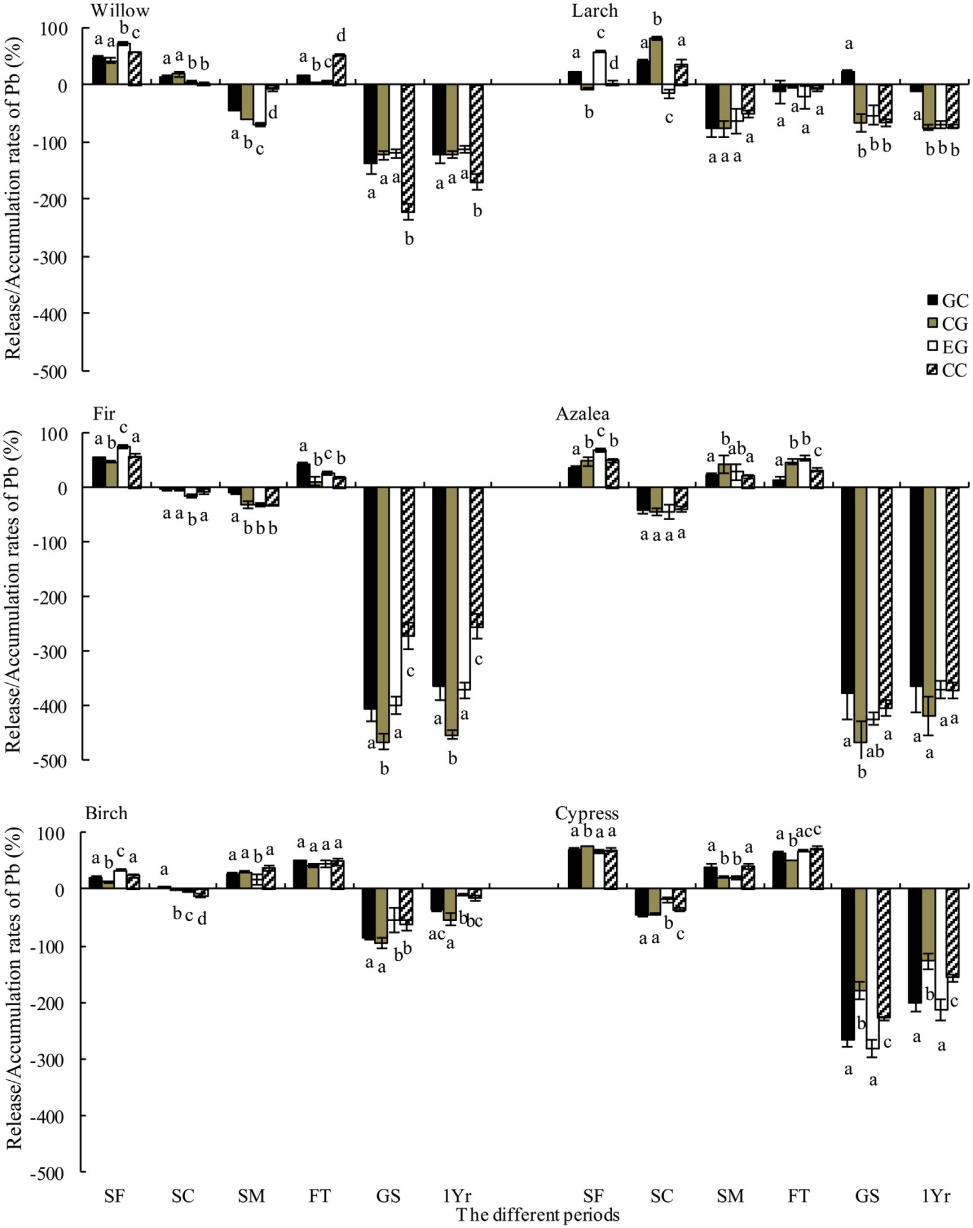
Release rate of Pb from foliar litter from gap center to closed canopy during different stages (mean ± SD, n = 3). FT, the freeze-thaw season; 1 Yr, one year. See Fig 3 for notes.

**Fig 6:**
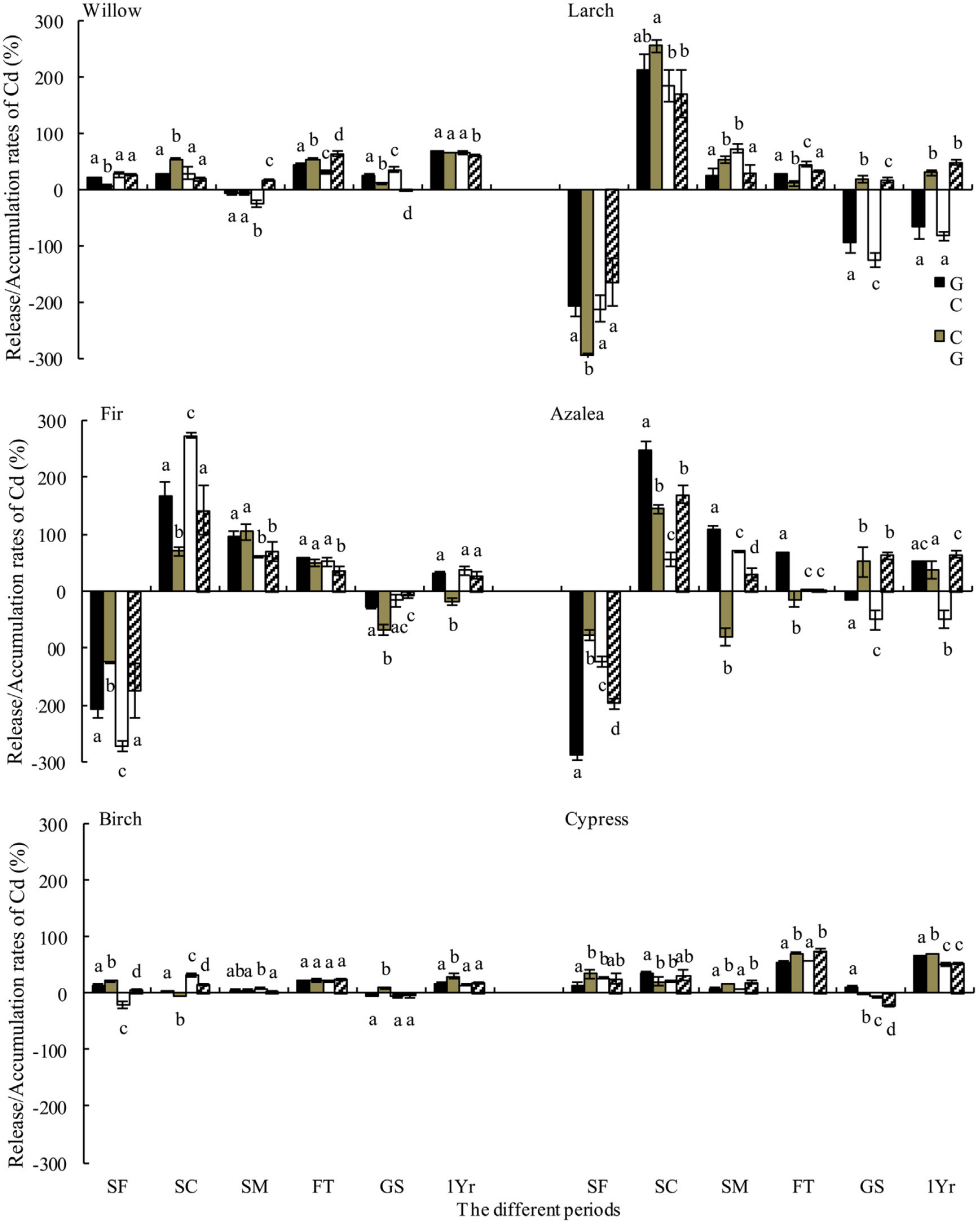
Release rate of Cd from foliar litter from gap center to closed canopy during different stages (mean ± *SD*, *n = 3*). FT, the freeze-thaw season; 1Yr, one year. See Fig 3 for notes.

Over one year of litter decomposition, Pb accumulated in the willow, larch, fir, azalea, birch and cypress litter and was especially prevalent in fir and azalea litter. Furthermore, Cd was released from decomposing litter regardless of the species (Fig 5 and Fig 6). The highest Pb accumulation rates in willow, larch and cypress litter were recorded at the extended gap edge and under the closed canopy (Fig 5). A similar pattern of Pb dynamics was observed in the growing season. The Cd release rates from willow, birch and cypress litter were higher in the gap center and at the canopy gap edge (Fig 6).

Although Pb was released from willow, fir, azalea, birch and cypress litter, it accumulated in the larch litter throughout the winter. The highest release rates from the willow, azalea and cypress litter were recorded at the extended gap edge and under the closed canopy (Fig 5). Furthermore, Cd was released from all six litter types during the winter, with the highest release rates in willow, larch, birch and cypress at the extended gap edge and under the closed canopy (Fig 6). The accumulation of Cd in the larch, birch and cypress litter was higher at the extended gap edge and under the closed canopy during the growing season.

During the snow formation stage, Pb was released from all species, whereas Cd was released from willow, birch and cypress litter and accumulated in the larch, fir and azalea litter (Fig 5 and Fig 6). The Pb release rates were greater at the extended gap edge and under the closed canopy for all species except cypress (Fig 5). The Cd accumulation rates for birch and cypress litter were greater in the gap center and at the canopy gap edge (Fig 6). During the snow coverage stage, Pb was released from willow and larch litter but accumulated in other litter. In contrast, Cd was released from the litter of all species. The highest Pb accumulation rates in fir and birch litter and the highest Cd release rates in fir and azalea litter were recorded at the extended gap edge and under the closed canopy (Fig 5 and Fig 6). During the snowmelt stage, the Pb concentration accumulated in willow, larch and fir litter and was released from the litter of other species. In contrast, Cd was released from all types of litter except for willow (Fig 5 and Fig 6). The accumulation rates of Pb from the willow and fir litter and the release rates from birch and cypress litter were greater at the extended gap edge and under the closed canopy (Fig 5). The Cd release rates from the larch, birch and cypress litter were greater at the extended gap edge and under the closed canopy (Fig 6).

### Correlations and Two-Way ANOVA

The daily mean temperature was highly and negatively correlated with the Pb release rate, but no significant correlations were found between the Cd release rate in the larch, fir, or azalea litter (Table 2). Table 3 shows that gap, species and their interactions significantly affected the dynamics of Pb and Cd during different decomposition periods.

**Table 2:** The correlation coefficients of metal release/accumulation rates of the experimental species leaf litter and litter average temperature (n = 48).

	Willow	Larch	Fir	Azalea	Birch	Cypress
Release/accumulation rates of Pb	-0.920**	-0.557**	-0.922**	-0.859**	-0.771**	-0.829**
Release/accumulation rates of Cd	-0.249	-0.109	-0.034	0.068	-0.343*	-0.790**

Significance level:

*, p<0.05;

**, p<0.01.

**Table 3:** Summary of two-way ANOVA on the effects of gap and species on Pb and Cd release/accumulation rates at different stages (*n = 72*).

	SF	SC	SM	FT	GS	1 Yr
	Pb					
Gap	247.429**	37.129**	15.409**	6.900**	6.636**	12.681**
Species	538.073**	447.673**	306.510**	151.388**	846.203**	857.916**
Gap×Species	40.276**	40.657**	8.079**	11.705**	23.210**	19.416**
	Cd					
Gap	19.566**	8.236**	28.906**	26.177**	61.686**	92.727**
Species	680.266**	283.181**	191.449**	187.268**	88.192**	204.836**
Gap×Species	35.523**	28.705**	68.056**	42.837**	57.387**	67.993**

Significance level:

**, *p*<0.01.

SF, snow formation stage; SC, snow coverage stage; SM, snow melt stage; FT, the freeze-thaw season; GS, growing season; 1 Yr, one year.

## Discussion

In contrast with our first hypothesis that more Pb and Cd are released from decomposing litter in the gap center than under the closed canopy, the highest rates of Pb and Cd release were recorded at the extended gap edge and under the closed canopy throughout the winter. Our data support the second hypothesis and indicate that the accumulation rates of Pb and Cd from the decomposing litter were greater at the extended gap edge and under the closed canopy than in the gap center and at the canopy gap edge. This result occurred because of the suitable hydrothermal environment during the growing season. The final hypothesis was that Cd release occurs more readily than that of Pb, which was also supported by our results. Overall, our results revealed that forest gaps had important implications for Pb and Cd dynamics during foliar litter decomposition in the alpine forest. In addition, litter quality and different stages of decomposition were associated with the release rates of Pb and Cd.

Many factors can influence metals dynamics, with the most important factors being environmental conditions, soil organisms, litter quality and the chemical properties of elements [34, 35]. The mechanisms that influence the release of metals are quite complex and can lead to variations in the Pb and Cd concentrations among species at different stages of decomposition. The obtained results suggest that the Pb concentrations in the litter increased significantly and that the Cd concentrations decreased after one year of decomposition. High concentrations of Pb that were observed in the litter correspond with the findings reported in other studies [36–38]. The observed dynamics of Cd are similar to the results of other experiments [37, 39]. Increases in the Pb and Cd concentrations in decomposing litter could be attributed to the following factors: (1) contamination by airborne particulates, (2) passive sorption onto recalcitrant organic fractions, (3) active accumulation by microbial colonizers and (4) the loss of leaf material, which decreases the litter weight and increases the mass-based metal concentration (assuming that the mass of the metal does not change) [36, 40]. In addition, the soluble components are released gradually as the decomposition process proceeds [41, 42]; therefore, litter residues contain significant amounts of Pb during the growing season. Decreased Cd concentrations could result from biological fixation and leaching [13, 43]. When the element concentration in the litter is higher than in the environment, the element would be released into the environment until equilibrium is reached.

In our research, similar patterns were observed for the Pb and Cd concentrations in the decomposing litter from the gap center and under the closed canopy during the winter. However, differences were observed during the growing season. The Pb and Cd concentrations in the foliar litter were higher in the gap center and at the canopy gap edge than at the extended gap edge and under the closed canopy during different periods of winter. This trend was similar to the trend observed for Pb during the growing season. Frequent freeze-thaw cycles and greater soil organism quantities and activities, among others, are important factors that affect Pb and Cd dynamics. Forest gaps change the distribution of snow in the winter and the hydrothermal conditions during the growing season, therefore, accounting for changes in the Pb and Cd concentrations in our study.

The Pb and Cd concentrations accumulated during the first year of decomposition in several previous studies [44, 45]. Unlike previous researchers, we found that Pb and Cd were released from litter throughout the winter and accumulated during the growing season. In practice, various factors influence heavy metal accumulation and release. First, the initial concentrations of the elements determined their release rates during the first year but had no influence on the concentrations or release rates during the later stages of needle litter decay [46]. During the snow formation stage, the release of Pb and Cd depended on the initial Pb and Cd concentrations. Second, soil organisms could play a very important role in metal cycling because they can mobilize or immobilize metals [47, 48]. Some microbial products, such as exopolymeric substances, can play a significant role in the sorption and transport of metals [49]. The insulating effects of snow result in greater soil organism activity, which is one reason why Cd was released from foliar litter during the snow coverage stage and the snow melt stage. Stronger leaching during the snowmelt stage could also explain the release of Cd [7, 50]. In addition, Pb was accumulated in fir, azalea, birch and cypress litter during the snow coverage stage and accumulated in the willow, larch and fir litter during the snow melt stage. Soil organisms immobilize metals for growth and production, while metals exceeding the needs of decomposers can accumulate during litter decomposition [51]. In addition, the natural organic matter can influence metal accumulation by forming strong metal complexes [52, 53]. Finally, previous studies have indicated that moisture and temperature can affect litter decomposition by changing the microenvironment and accelerating the shifts of microbial assemblages from inactive to fully active [54, 55]. Because sufficient moisture, a warmer environment and more soil organisms were present during the growing season, Pb and Cd accumulated in the decomposing litter. This correlation also demonstrates that the distribution of snow and the hydrothermal differences caused by the gaps could significantly impact the Pb and Cd release and that the Cd dynamics of the larch, fir, and azalea litter are substantially influenced by litter quality.

The accumulation and release dynamics of heavy metals in litter were divergent from the gap center to the closed canopy. In this study, the litter at the extended gap edge and under the closed canopy released and accumulated metals at higher rates than in the gap center or at the canopy gap edge during the winter and the growing season, respectively. Strong freezing, thawing and freeze-thaw cycles at the extended gap edge with thin snow cover and closed canopy with none snow patches could promote heavy metals release from litter in winter. In contrast, there are greater numbers and activity of soil organisms resulting in the greater release of heavy metals through biological processes during the growing season. The observations here agree with the previous study, which documented there were different control factors in litter decomposition between in winter (freeze-thaw and other physical effects) and in growing season (decomposers activity) [56]. The higher accumulation/release rates at the extended gap edge and under the closed canopy may also be explained by the following reasons. First, foliar litter decomposes faster under appropriate hydrothermal conditions [55], and repeated freeze-thaw cycles in the winter could promote litter decomposition by changing the quality of the organic matter [19]. Consequently, the soluble components are released from the litter [42], but the recalcitrant components, such as Pb and Cd, remain. Second, the higher Pb and Cd accumulation rates may be explained by the large amounts of Pb and Cd that are released during the winter. The resulting lack of metals could promote immobilization by organisms rather than release from litter. Finally, humic and fulvic acids absorb various metal ions via chelation, this process results in the release of Pb and Cd at the extended gap edge and under the closed canopy. In summary, our results reveal that the dynamics of Pb are significantly influenced by the frequent freeze-thaw cycles in winter and the suitable hydrothermal environment during the growing season, and the dynamics of Cd are mostly influenced by litter quality and soil organism.

## Conclusions

Our results indicate that freeze-thaw cycles in winter and the suitable hydrothermal environment during the growing season at the extended gap edge and under the closed canopy could promote the release of Pb during foliar litter decomposition. The lower temperatures at the extended gap edge and under the closed canopy were more favorable for releasing Cd, whereas most litter species were more affected by physico-chemical properties. Therefore, although the dynamics of Cd release were largely related to litter quality, the decreased snow depth in the winter warming scenario would promote the release of Pb during litter decomposition in the alpine forest. Pb and Cd release/accumulation rates from foliar litter were higher at the extended gap edge and under the closed canopy than in the gap center and at the canopy gap edge. Overall, forest gap formation may inhibit biogeochemical cycling of Pb and Cd. These results highlight the importance of forest gaps for Pb and Cd dynamics during the initial year of litter decomposition in the alpine forest. To clarify the specific mechanisms of Pb and Cd released under the influence of forest gaps, more focused and long-term research is required.

## Supporting Information

S1 Dataset Dataset used in the present study. (XLSX)

## References

[1] Tagliavini M, Tonon G, Scandellari F, Quinones A, Palmieri S, Menarbin G, et al Nutrient recycling during the decomposition of apple leaves (Malus domestica) and mowed grasses in an orchard. Agriculture, ecosystems & environment. 2007;118(1):191–200.

[2] Cáliz J, Montserrat G, Martí E, Sierra J, Chung AP, Morais PV, et al Emerging resistant microbiota from an acidic soil exposed to toxicity of Cr, Cd and Pb is mainly influenced by the bioavailability of these metals. Journal of Soils and Sediments. 2013;13(2):413–28.

[3] Giller KE, Witter E, Mcgrath SP. Toxicity of heavy metals to microorganisms and microbial processes in agricultural soils: a review. Soil Biology and Biochemistry. 1998;30(10):1389–414.

[4] Brokaw N, Busing RT. Niche versus chance and tree diversity in forest gaps. Trends in ecology & evolution. 2000;15(5):183–8. 10782131 10.1016/s0169-5347(00)01822-x

[5] Stan AB, Daniels LD. Growth releases across a natural canopy gap-forest gradient in old-growth forests. Forest Ecology and Management. 2014;313:98–103.

[6] He W, Wu FZ, Yang WQ, Wu QQ, He M, Zhao YY. Effect of snow patches on leaf litter mass loss of two shrubs in an alpine forest. Chinese Journal of Plant Ecology. 2013;37(4):306–16.

[7] Tan B, Wu F, Yang W, He X. Snow removal alters soil microbial biomass and enzyme activity in a Tibetan alpine forest. Applied Soil Ecology. 2014;76:34–41.

[8] Zhu JX, He XH, Wu FZ, Yang WQ, Tan B. Decomposition of Abies faxoniana litter varies with freeze–thaw stages and altitudes in subalpine/alpine forests of southwest China. Scandinavian Journal of Forest Research. 2012;27(6):586–96.

[9] Batty LC, Younger PL. The effect of pH on plant litter decomposition and metal cycling in wetland mesocosms supplied with mine drainage. Chemosphere. 2007;66(1):158–64. 16820189 10.1016/j.chemosphere.2006.05.039

[10] Laskowski R, Berg B. Dynamics of some mineral nutrients and heavy metals in decomposing forest litter. Scandinavian Journal of Forest Research. 1993;8(1–4):446–56.

[11] Edmonds RL, Tuttle KM. Red alder leaf decomposition and nutrient release in alder and conifer riparian patches in western Washington, USA. Forest Ecology and Management. 2010;259(12):2375–81.

[12] Saccone P, Morin S, Baptist F, Bonneville JM, Colace MP, Domine F, et al The effects of snowpack properties and plant strategies on litter decomposition during winter in subalpine meadows. Plant and soil. 2013;363(1–2):215–29.

[13] Lemma B, Nilsson I, Kleja DB, Olsson M, Knicker H. Decomposition and substrate quality of leaf litters and fine roots from three exotic plantations and a native forest in the southwestern highlands of Ethiopia. Soil Biology and Biochemistry. 2007;39(9):2317–28.

[14] Christenson LM, Mitchell MJ, Groffman PM, Lovett GM. Winter climate change implications for decomposition in northeastern forests: comparisons of sugar maple litter with herbivore fecal inputs. Global Change Biology. 2010;16(9):2589–601.

[15] Freppaz M, Williams BL, Edwards AC, Scalenghe R, Zanini E. Simulating soil freeze/thaw cycles typical of winter alpine conditions: implications for N and P availability. Applied Soil Ecology. 2007;35(1):247–55.

[16] Conn C, Dighton J. Litter quality influences on decomposition, ectomycorrhizal community structure and mycorrhizal root surface acid phosphatase activity. Soil Biology and Biochemistry. 2000;32(4):489–96.

[17] Leroy CJ, Marks JC. Litter quality, stream characteristics and litter diversity influence decomposition rates and macroinvertebrates. Freshwater biology. 2006;51(4):605–17.

[18] Yang XD, Chen J. Plant litter quality influences the contribution of soil fauna to litter decomposition in humid tropical forests, southwestern China. Soil Biology and Biochemistry. 2009;41(5):910–8.

[19] Schmitt A, Glaser B, Borken W, Matzner E. Repeated freeze–thaw cycles changed organic matter quality in a temperate forest soil. Journal of Plant Nutrition and Soil Science. 2008;171(5):707–18.

[20] Groffman PM, Driscoll CT, Fahey TJ, Hardy JP, Fitzhugh RD, Tierney GL. Effects of mild winter freezing on soil nitrogen and carbon dynamics in a northern hardwood forest. Biogeochemistry. 2001;56(2):191–213.

[21] Carlson DW, Groot A. Microclimate of clear-cut, forest interior, and small openings in trembling aspen forest. Agricultural and Forest Meteorology. 1997;87(4):313–29.

[22] Muscolo A, Sidari M, Mercurio R. Influence of gap size on organic matter decomposition, microbial biomass and nutrient cycle in Calabrian pine (Pinus laricio, Poiret) stands. Forest Ecology and Management. 2007;242(2):412–8.

[23] Arunachalam A, Arunachalam K. Influence of gap size and soil properties on microbial biomass in a subtropical humid forest of north-east India. Plant and soil. 2000;223(1–2):187–95.

[24] Covelo E, Vega F, Andrade M. Simultaneous sorption and desorption of Cd, Cr, Cu, Ni, Pb, and Zn in acid soils: I. Selectivity sequences. Journal of hazardous materials. 2007;147(3):852–61. 17346879 10.1016/j.jhazmat.2007.01.123

[25] Vorobeichik E, Pishchulin P. Effect of individual trees on the pH and the content of heavy metals in forest litters upon industrial contamination. Eurasian Soil Science. 2009;42(8):861–73.

[26] Yang WQ, Wang KY, Kellomäki S, Zhang J. Annual and monthly variations in litter macronutrients of three subalpine forests in western China. Pedosphere. 2006;16(6):788–98.

[27] Liu JL, Wu FZ, Yang WQ, Shi PL, Wang A, Yang YL, et al Effect of seasonal freeze–thaw cycle on net nitrogen mineralization of soil organic layer in the subalpine/alpine forests of western Sichuan, China. Acta Ecologica Sinica. 2013;33(1):32–7.

[28] Tan B, Wu FZ, Yang WQ, Liu L, Yu S. Characteristics of soil animal community in the subalpine/alpine forests of western Sichuan during onset of freezing. Acta Ecologica Sinica. 2010;30(2):93–9.

[29] Schliemann SA, Bockheim JG. Methods for studying treefall gaps: a review. Forest Ecology and Management. 2011;261(7):1143–51.

[30] Wu QG, Wu FZ, Yang WQ, Tan B, Yang YL, Ni XY, et al Characteristics of gaps and disturbance regimes of the alpine fir forest in western Sichuan. Chinese Journal of Applied Environmental Biology. 2013;19(6):922–8.

[31] Kaila A, Asam ZZ, Sarkkola S, Xiao L, Laurén A, Vasander H, et al Decomposition of harvest residue needles on peatlands drained for forestry–Implications for nutrient and heavy metal dynamics. Forest Ecology and Management. 2012;277:141–9.

[32] Lu R. Soil and agro-chemical analytical methods. China Agricultural Science and Technology Press, Beijing 1999:146–95.

[33] Jacob M, Weland N, Platner C, Schaefer M, Leuschner C, Thomas FM. Nutrient release from decomposing leaf litter of temperate deciduous forest trees along a gradient of increasing tree species diversity. Soil Biology and Biochemistry. 2009;41(10):2122–30.

[34] Aerts R. Climate, leaf litter chemistry and leaf litter decomposition in terrestrial ecosystems: a triangular relationship. Oikos. 1997:439–49.

[35] Yang WQ, Deng RJ, Zhang J. Forest litter decomposition and its responses to global climate change. The journal of applied ecology. 2007;18(12):2889–95. 18333472

[36] Du Laing G, Ryckegem GV, Tack FM, Verloo MG. Metal accumulation in intertidal litter through decomposing leaf blades, sheaths and stems of Phragmites australis. Chemosphere. 2006;63(11):1815–23. 16330074 10.1016/j.chemosphere.2005.10.034

[37] Lehto T, Smolander A, Aphalo PJ. Decomposition and element concentrations of silver birch leaf litter as affected by boron status of litter and soil. Plant and soil. 2010;329(1–2):195–208.

[38] Windham L, Weis JS, Weis P. Metal dynamics of plant litter of Spartina alterniflora and Phragmites australis in Metal‐Contaminated salt marshes. Part 1: Patterns of decomposition and metal uptake. Environmental Toxicology and Chemistry. 2004;23(6):1520–8. 15376538 10.1897/03-284

[39] Pereira P, Caçador I, Vale C, Caetano M, Costa AL. Decomposition of belowground litter and metal dynamics in salt marshes (Tagus Estuary, Portugal). Science of the Total Environment. 2007;380(1):93–101. 17316771 10.1016/j.scitotenv.2007.01.056

[40] Inman JC, Parker G. Decomposition and heavy metal dynamics of forest litter in northwestern Indiana. Environmental Pollution (1970). 1978;17(1):39–51.

[41] Baptist F, Yoccoz NG, Choler P. Direct and indirect control by snow cover over decomposition in alpine tundra along a snowmelt gradient. Plant and soil. 2010;328(1–2):397–410.

[42] Wu FZ, Yang WQ, Zhang J, Deng RJ. Litter decomposition in two subalpine forests during the freeze–thaw season. Acta Oecologica. 2010;36(1):135–40.

[43] Hutchins S, Davidson M, Brierley J, Brierley C. Microorganisms in reclamation of metals. Annual Reviews in Microbiology. 1986;40(1):311–36. 10.1146/annurev.mi.40.100186.001523 3535647

[44] Brun CB, Åström ME, Peltola P, Johansson M-B. Trends in major and trace elements in decomposing needle litters during a long-term experiment in Swedish forests. Plant and soil. 2008;306(1–2):199–210.

[45] Laskowski R, Niklińska M, Maryański M. The dynamics of chemical elements in forest litter. Ecology. 1995:1393–406.

[46] Berg B, Staaf H. Decomposition rate and chemical changes of Scots pine needle litter. II. Influence of chemical composition. Ecological Bulletins. 1980;32:373–90.

[47] Gadd GM. Microbial influence on metal mobility and application for bioremediation. Geoderma. 2004;122(2):109–19.

[48] Burgos A, Maldonado J, De los Rios A, Solé A, Esteve I. Effect of copper and lead on two consortia of phototrophic microorganisms and their capacity to sequester metals. Aquatic Toxicology. 2013;140:324–36. 10.1016/j.aquatox.2013.06.022 23891781

[49] Kantar C, Demiray H, Dogan NM. Role of microbial exopolymeric substances (EPS) on chromium sorption and transport in heterogeneous subsurface soils: II. Binding of Cr (III) in EPS/soil system. Chemosphere. 2011;82(10):1496–505. 10.1016/j.chemosphere.2010.11.001 21094978

[50] Elliott J. Evaluating the potential contribution of vegetation as a nutrient source in snowmelt runoff. Canadian Journal of Soil Science. 2013;93(4):435–43.

[51] Heinlaan M, Ivask A, Blinova I, Dubourguier H-C, Kahru A. Toxicity of nanosized and bulk ZnO, CuO and TiO to bacteria Vibrio fischeri and crustaceans Daphnia magna and Thamnocephalus platyurus. Chemosphere. 2008;71(7):1308–16. 10.1016/j.chemosphere.2007.11.047 18194809

[52] Rashid M. Absorption of metals on sedimentary and peat humic acids. Chemical Geology. 1974;13(2):115–23.

[53] Pesch ML, Hoffmann M, Christl I, Kraemer S, Kretzschmar R. Competitive ligand exchange between Cu–humic acid complexes and methanobactin. Geobiology. 2013;11(1):44–54. 10.1111/gbi.12010 23082815

[54] Pu G, Du J, Ma X, Lv Y, Jia Y, Jia X, et al Contribution of ambient atmospheric exposure to Typha angustifolia litter decomposition in aquatic environment. Ecological Engineering. 2014;67:144–9.

[55] Zhang DQ, Hui DF, Luo YQ, Zhou GY. Rates of litter decomposition in terrestrial ecosystems: global patterns and controlling factors. Journal of Plant Ecology. 2008;1(2):85–93.

[56] Zhu JX, Yang WQ, He XH. Temporal dynamics of abiotic and biotic factors on leaf litter of three plant species in relation to decomposition rate along a subalpine elevation gradient. PloS one. 2013;8(4):e62073 10.1371/journal.pone.0062073 23620803 PMC3631152

